# Caterpillar-Induced Volatile Emissions in Cotton: The Relative Importance of Damage and Insect-Derived Factors

**DOI:** 10.3389/fpls.2021.709858

**Published:** 2021-08-03

**Authors:** Carla M. Arce, Gaia Besomi, Gaétan Glauser, Ted C. J. Turlings

**Affiliations:** ^1^Laboratory of Fundamental and Applied Research in Chemical Ecology, Institute of Biology, University of Neuchâtel, Neuchâtel, Switzerland; ^2^Neuchâtel Platform of Analytical Chemistry, University of Neuchâtel, Neuchâtel, Switzerland

**Keywords:** cotton, *Spodoptera* spp., plant indirect defenses, volatile emissions, herbivore-associated molecular patterns, damage-associated molecular patterns

## Abstract

In response to herbivore attack, plants release large amounts of volatiles that can serve as attractants for the natural enemies of the attacking herbivores. Such responses are typically triggered by damage- and insect-associated factors. Cotton plants are somewhat peculiar because they release specific blends of volatiles in two waves in response to caterpillar attack. They first emit constitutively stored volatile compounds, and after about 24 h a second wave that includes various *de novo* synthesized compounds. The relative importance of damage-associated and insect associated-factors in this induction of cotton volatile emissions is not yet fully clear. We evaluated how cotton plants respond to mechanical damage and to the application of the oral secretion from the generalist lepidopteran pest *Spodoptera exigua*, by measuring the local and systemic emissions of volatile compounds from their leaves. Our results confirm that cotton plants respond to damage-associated molecular patterns (DAMPs) as well as to herbivore-associated molecular patterns (HAMPs) present in the caterpillars’ oral secretion. Interestingly, a stronger response was observed for cotton plants that were treated with oral secretion from cotton-fed caterpillars than those fed on maize. We tested the possibility that volicitin, a common fatty acid-derived elicitor in caterpillar regurgitant plays a role in this difference. Volicitin and volicitin-like compounds were detected in equal amounts in the oral secretion of *S. exigua* fed on either cotton or maize leaves. We conclude that other elicitors must be involved. The identification of these eliciting cues is expected to contribute to the development of novel strategies to enhance the resistance of cotton plants to insect pests.

## Introduction

During the millions of years of interaction with herbivorous insects, plants have evolved a multitude of defense strategies. These include constitutive defenses as well as induced defenses, which are produced only upon herbivore attack (Karban and Baldwin, 1997; Farmer, 2014; Erb and Reymond, 2019). Among the inducible defenses are so-called “herbivore-induced plant volatiles” (HIPVs), that can serve as foraging cues for natural enemies of herbivores (Dicke et al., 1988; Turlings et al., 1990; Paré and Tumlinson, 1999; Turlings and Wäckers, 2004; Dicke and Baldwin, 2010; Heil and Land, 2014; Aljbory and Chen, 2018; Turlings and Erb, 2018).

Plants are able to distinguish between herbivory and mere mechanical damage. This ability allows plants to avoid wasting valuable resources for defenses in situations where they are not needed. Damage itself is a key factor by which plants recognize that they are under attack (Heil, 2009), but alone it is not sufficient to trigger full plant defense responses (Fürstenberg-Hägg et al., 2013; Acevedo et al., 2015; Schmelz, 2015). This is also the case for HIPVs (Turlings et al., 1990; Dicke, 2016; Turlings and Erb, 2018). While feeding, herbivores introduce molecules from their oral secretion into plant tissue, which can lead to the release of endogenous signal molecules from disrupted cells (Acevedo et al., 2015; Duran-Flores and Heil, 2016; Erb and Reymond, 2019). Hence, plants rely on the recognition of non-self elicitors and damaged-self associated molecules to launch appropriate defense responses to an attack. These insect-derived elicitors are known as herbivore-associated molecular patterns (HAMPs), whereas plant-derived inducers are known as damage-associated molecular patterns (DAMPs). HAMPs can be found in the oral secretions and oviposition fluids of insects (Felton and Tumlinson, 2008; Wu and Baldwin, 2009). After perceiving the attacker, the plant activates downstream signaling mechanisms, resulting in activation of the immune system. To date, various elicitors in insect oral secretions have been shown to play a role in the recognition of herbivore attackers by plants (Turlings et al., 1993, 2000; Mattiacci et al., 1995; Alborn et al., 1997, 2007; Schmelz et al., 2006; Louis et al., 2013; Acevedo et al., 2015; Basu et al., 2018). Specific examples of caterpillar-produced HAMPs include β-glucosidase, found in the oral secretion of *Pieris brassicae* fed on cabbage, which triggers the release of volatiles that attract the parasitic wasp *Cotesia glomerata* (Mattiacci et al., 1994, 1995), volicitin, which is a fatty acid-derived elicitor, first isolated from *Spodoptera exigua* caterpillars fed on maize plants (Alborn et al., 1997; Turlings et al., 2000), and inceptin, a peptide found in the oral secretions of *Spodoptera* caterpillars after they ingest chloroplast-containing plant tissue (Schmelz et al., 2006, 2009).

Damage-associated molecular patterns, the second category of signal molecules, are the basis for the mechanism of plant self-recognition (Green and Ryan, 1972; Heil, 2009; Duran-Flores and Heil, 2016). They are endogenous plant-derived indicators of injury, which are released from damaged cells into the extracellular space (Lotze et al., 2007; Heil and Land, 2014; Choi and Klessig, 2016; Gust et al., 2017). The signals generated upon herbivory allow injured cells to communicate their damage status to other cells, thereby activating downstream signaling defense mechanisms (Henry et al., 2012). DAMPs comprise molecules such as cell wall components, fragmented DNA, ATP, and peptides (Quintana-Rodriguez et al., 2018; Hou et al., 2019). A well-studied DAMP is systemin, a polypeptide formed in tomato leaves and perceived by the plant as a primary signal for systemic defense responses (Pearce et al., 1991; Pearce and Ryan, 2003). Such peptides are released by damaged cells and trigger an immune response in the plant (Huffaker et al., 2006, 2013; Yamaguchi et al., 2006). Several studies have demonstrated the effects of DAMPs by applying plant extracts to several species of plants, which resulted in enhanced plant resistance (Mattiacci et al., 1995; Devaiah et al., 2009; Quintana-Rodriguez et al., 2018). In recent years, it has been suggested that the combined recognition of DAMPs and HAMPs, rather than single molecules, provides plants with specific information regarding the nature of an ongoing attack (Huffaker and Ryan, 2007; Duran-Flores and Heil, 2016).

Cotton (*Gossypium hirsutum*) is a plant of great economic importance. It is known to have direct defenses such as gossypol, a terpenoid with insecticidal properties, and indirect defenses such as extra-floral nectar and HIPVs (Loughrin et al., 1994; McCall et al., 1994; McAuslane et al., 1997; Röse and Tumlinson, 2004; Rose et al., 2006). Despite these defenses, cotton plants are subject to attack by rich and complex groups of arthropod herbivores, and are known to be one of the “dirtiest” crops in the world because of the large quantities of pesticides used against these pests (Naranjo et al., 2008; Hagenbucher et al., 2013). The moth *S. exigua* is a widely distributed polyphagous pest of numerous cultivated crops, including cotton plants (Eveleens et al., 1973; Greenberg et al., 2001; Zheng et al., 2011). Previous studies have shown that cotton releases constitutively stored volatiles immediately following attack by *S. exigua* caterpillars, whereas several other volatiles are *de novo* synthesized and only emitted after more than 24 h of feeding damage (Loughrin et al., 1994; McCall et al., 1994). These truly inducible volatiles can be systemically released, and are also released from undamaged parts of the plant (Paré and Tumlinson, 1998; Röse and Tumlinson, 2005). Paré and Tumlinson (1997) found that cotton plants treated with *S. exigua* oral secretion released higher amounts of volatiles than plants that had only been mechanically damage.

Only a handful of elicitors have been identified so far, yet considering the vast number of herbivorous insect species and the plants they feed on, more can be expected to be involved in mediating plant-insect interactions (Bonaventure et al., 2011). No specific elicitor has been identified from insect oral secretions that trigger defense responses in cotton leaves. From previous studies (described above), we surmise that it is likely that cotton is able to perceive elicitor-like molecules from the caterpillars that feed on them, but is unclear to what extent DAMPs and HAMPs jointly contribute to the responses in cotton plants. We tested this by measuring local and systemic HIPV emissions in cotton plants after different treatments with the regurgitant (R) of *S. exigua* caterpillars fed on different plants. We also measured the amount of the known elicitor volicitin and volicitin-like compounds in the caterpillars regurgitant. The results indicate that the inducible responses in cotton are driven by HAMPs as well as DAMPs, and that the oral secretion of cotton-fed caterpillars is particularly active.

## Materials and Methods

### Plants

Cultivated cotton seeds (*G. hirsutum* L., var. STAM 59A) were obtained from CIRAD (La recherche agronomique pour le développement, Montpellier, France). Seeds were soaked in tap water and covered with aluminum foil for 24 h at 24°C. Subsequently, they were kept in plastic boxes with a 4-cm layer of moist vermiculite until germination. Around 4 days after germination, the seedlings were transplanted to individual plastic pots filled with plant substrate soil (Profi Substrat soil, Einheitserde, Germany). The plants were grown under greenhouse conditions (L16:D8, T = 30°C ± 5, and R.H. = 60–80%) and watered every 2 days. All the plants used in the experiments had either two or four fully developed leaves. Maize seeds (*Zea mays* L., var. Delprim) were purchased from DSP Delley seeds and plants genetics Ltd., (Delley, Switzerland) and they were germinated in individual plastic pots filled with substrate soil (Profi Substrat soil, Einheitserde, Germany) and kept under greenhouse conditions (as above). The plants used in the experiments were 10 days old.

### Insects

*Spodoptera exigua* (Hübner; Lepidoptera; Noctuidae) eggs were purchased from Entocare (Wageningen, Netherlands). The caterpillars were reared on wheat-germ based artificial diet (Frontier Scientific Services, Newark, United States) under laboratory conditions (25 ± 2°C, 60% relative humidity, 16:8 h L/D). Regurgitant (R) was collected according to Turlings et al. (1993). Briefly, third to fourth instar caterpillars were gently squeezed close to the head, which forced them to regurgitate (about 10 μl per caterpillar), which was collected through 25 μl capillaries inserted into a 3-ml vial and attached to a vacuum pump (low pressure). The caterpillars were previously fed on either cotton or maize leaves for 24 h. The regurgitant samples were stored at −80°C until further use.

### Local and Systemic Induction of Cotton Plants by Simulating Herbivory

**Figure 1:**
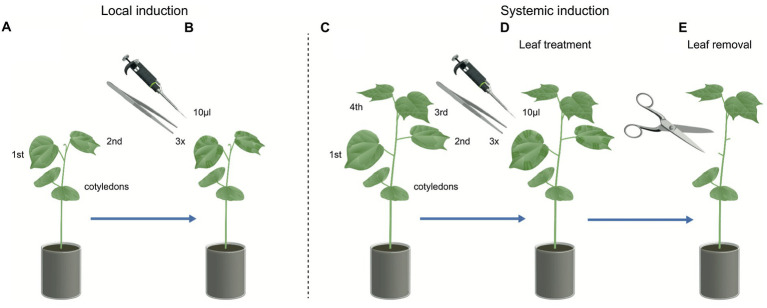
Illustration of simulated herbivory on cotton plants. Local induction: **(A)** Two-leaves stage cotton plants used to measure local herbivore-induced plant volatile (HIPV) emissions. **(B)** The first and second leaf were wounded three times with a serrated forceps and immediately afterward 10 μl of regurgitant were applied on the wounded sites, the HIPVs were collected over time. Systemic induction: **(C)** Four-leaves stage cotton plants used to measure systemic HIPV emissions. **(D)** The first and second leaf were wounded three times with a serrated forceps and immediately afterward 10 μl of regurgitant were applied on the wounded sites. **(E)** To measure systemically induced volatiles emissions (from the third and fourth leaf), the treated first and second leaves were removed right before the volatile collection.

In order to test whether cotton plants respond specifically to HAMPs present in the regurgitant of *S. exigua* fed on cotton plants, we performed a series of experiments to measure local and systemic volatile emissions over time. First, we carried out an experiment with cotton plants that had two developed leaves and were induced by simulating caterpillar damage and applying regurgitant to the wounds (Figures 1A,B). In brief, we collected emitted volatiles from cotton plants (*n* = 4–5) that were either mechanically damaged (MD), were MD and with application of regurgitant from *S. exigua* fed on cotton plants (MD + CR) and were mechanically damaged and with application of regurgitant from *S. exigua* fed on maize plants (MD + MR). Control plants were not damaged. Mechanical damage was inflicted by scratching one leaf (~2 cm^2^) with a pair of serrated forceps. Three sites on each side of the leaf were damaged in this way, and 10 μl of regurgitant (the approximate equivalent to the volume collected per caterpillar) was immediately applied directly onto the wounded sites (Turlings et al., 1990, 1998; Erb et al., 2015; De Lange et al., 2020). The collection volatiles were performed 2 h after the first induction and repeated 24 and 48 h later. The induction treatments were repeated 2 h before each collection timing alternating the leaf damaged (De Lange et al., 2020).

To evaluate the importance of HAMPs in the systemic response of HIPVs in cotton plants, we collected emitted volatiles from plants induced with *S. exigua* regurgitant. Plants with four developed leaves were used and induced the same way as described above (mechanical damage and regurgitant application). However, this time the two lower leaves (1st and 2nd leaves) were treated, and after the final induction were removed, and the volatiles were collected only from the untreated 3rd and 4th leaves (Figures 1C–E). Cotton plants (*n* = 6) were induced twice a day (morning and evening) for 2 consecutive days. The plants were induced by MD, mechanical damage with regurgitant from *S. exigua* fed on cotton plants (MD + CR), and mechanical damaged with regurgitant from *S. exigua* fed on maize plants (MD + MR). Control plants were not damaged, but the two lower leaves were also removed. On the 3rd day, after 2 days of being induced by simulated herbivory, the plants received the last induction treatment at 8 am. The damaged leaves (1st and 2nd leaves) were removed 2 h later at 10 am, and the collection of systemic HIPVs was immediately performed.

Lastly, we used detached cotton and maize plants to incubate them in a regurgitant solution (Supplementary Figure 1), as the plants have to uptake the regurgitant to be induced. We could, therefore, evaluate the effect of the regurgitant itself. The incubation was performed according to Turlings et al. (2000). The plants were subjected to the following treatments: cotton and maize plants incubated in water, cotton plants incubated in regurgitant from *S. exigua* fed on cotton plants (CR), cotton plants incubated in regurgitant from *S. exigua* fed on maize plants (MR), and maize plants incubated in regurgitant from *S. exigua* fed on maize (MR). Based on the previous study, maize plants are known to respond to this form of induction, and (Turlings et al., 2000); that is why were used as a positive control. Plants were cut close to the soil and immediately placed in a 1.5-ml Eppendorf tube with 500 μl of 10% regurgitant solution (50 μl of filtered regurgitant in 450 μl of Milli-Q water). To avoid the regurgitant oxidation, the tubes were covered with aluminum foil. In order to prevent bacterial growth, the regurgitant was filtered (Turlings et al., 1993). To this end, the regurgitant samples were centrifuged for 20 min at 12,000 rpm and the supernatant was collected and filtered (13 mm Syringe filter, PTFE hydrophilic, 0.22 μm, BGB, Switzerland). The time course of the volatile collection was the same as described above (2, 24, and 48 h after induction). For this, we used three different batches of plants. The plants were incubated for 2 h before volatile collection (morning period). After the end of the volatile collection, plants were kept for the next 2 h in the regurgitant solution. In total, the plants were incubated in the regurgitant solution for 6 h. After that, plants were removed from the regurgitant solution and 0.5 cm of the stem was cut off. Plants were kept overnight in falcon tubes containing 10 ml of Milli-Q water. This procedure was performed to keep the plants hydrated during the entire experiment, which lasted for 48 h. The next day the same incubation treatment was carried out: incubation in regurgitant solution for 2 h prior to volatile collection, and then followed by the incubation in water overnight.

### Collection of Volatiles

Plants were carefully placed in one-port glass bottles to collect the volatiles as described by Turlings et al. (1993, 2000, 2004). The volatiles were collected using trapping filters containing 25 mg of 80/100 mesh Hayesep-Q adsorbent (Ohio Valley Specialist Company, Marietta, United States) for 2 h between 11 am and 1 pm. This period of the day is when cotton plants emit the highest amounts of volatiles (Rose et al., 1996). After each collection, the filters were eluted with 150 μl of dichloromethane (Honeywell, Riedel-de Haën, DE), and 10 μl of internal standard was added (*n*-octane and *n*-nonyl acetate, 20 ng/μl each; Turlings et al., 2000). The samples were stored at −80°C until further analyses.

### Analysis of Volatiles

The volatile samples were analyzed on a gas chromatograph (Agilent 7890B) coupled with a mass spectrometer (Agilent 5977B GC/MSD) on TIC mode. About 2 μl of sample were injected in pulsed splitless mode onto an Agilent HP-5MS column (30 m length x 0.25 mm diameter and 0.25 μm film thickness). The temperature program was as follows: kept at 40°C for 3 min, increased to 100°C at a rate of 8°C min^−1^ and subsequently at 5°C min^−1^ to 200°C, followed by a post run period of 3 min at 250°C. Helium was used as a carrier gas and kept at constant flow of 1.1 ml min^−1^. The identification and quantification of compounds were performed using comparisons to the mass spectra of commercial standards and NIST 17 library spectra.

### Volicitin Extraction and Analysis

In order to check whether *S. exigua* fed on cotton plants produce elicitors, we measured relative amounts of volicitin and volicitin-like compounds content in regurgitant of *S. exigua* caterpillars fed on either cotton or maize plants. FACs, specially volicitin *N*-(17-hydroxylinolenoyl)-L-glutamine were chosen based on previous results from Alborn et al. (2000) and Turlings et al. (2000), where it was shown that *S. exigua* fed on maize plants produce this elicitor, which is responsible for inducing HIPV emission in maize plants. To this end, the regurgitant was collected as previously described, each sample (*n* = 5) corresponded to 10 μl of regurgitant collected from two caterpillars. For the extraction, 1 ml of MeOH:H_2_O 50% (50:50) was added to each sample, which was then vortexed and centrifuged at 10,000 rpm and at 4°C for 15 min. The supernatant was collected and used for volicitin analysis.

The analysis of volicitin was performed by UHPLC MS instrument (Waters) made up with using ultra-high performance liquid chromatography quadrupole-time-of-flight mass spectrometry. Specifically, an Acquity UPLC (Waters) coupled to a Synapt G2 high-resolution mass spectrometer. QTOF was employed. The column used for separation was Acquitting UPLC BEH C18 1.7 μm, 2.1 × 50 mm (Waters). The temperature was maintained at 25°C. Two eluants were used: water and 0.05% of formic acid (eluant A) and acetonitrile and 0.05% of formic acid (eluant B). A linear gradient from 10 to 100% B in 7 min was applied. The injection volume was 2.5 μl. The mass spectrometer source was operated in electrospray negative ionization and data were acquired in data-independent acquisition (DIA) mode (so-called MSe which alternates between low and high collision energies). Exact mass measurements (<2 ppm) were ensured by infusing a 500 ng/ml solution of leucine-enkephalin at 15 μl/min through the Lockspray probe. For data acquisition and processing, we used the software Masslynx v.4.1 (Waters). Volicitin and related molecules were identified based on the determination of the most probable molecular formula as well as fragmentation pattern (typical fragment at *m/z* 145.0615 corresponding to a glutamine moiety). Peaks corresponding to volicitin were volicitins were automatically integrated using TargetLynx XS with a 0.1 min chromatographic window centered on the retention time of each compound and a 0.02 Da mass window centered on the (M-H)^−^ ion.

### Statistical Analysis

Statistical tests were carried out in R (v. 4.0.0; Team, 2001) using Analysis of Deviance (ANODEV; a maximum likelihood equivalent of ANOVA), followed by residual analysis to verify suitability of distributions of the tested models. Generalized Linear Models (GLM) with a Gaussian distribution were used to verify the differences in volatile emissions and volicitin. Least Squares Means (*LSMeans*) were used to compare significant differences among treatments. An orthogonal partial least squares discriminant analysis (OPLS-DA) and hierarchical clustering heatmap were carried using MetaboAnalyst 5.0 (Wiklund et al., 2008; Chong et al., 2019) to check for differences among treatments on local and systemic HIPVs profiles after 48 h.

## Results

### Cotton Plants Induced Local and Systemic HIPV After Simulated Herbivory

**Figure 2:**
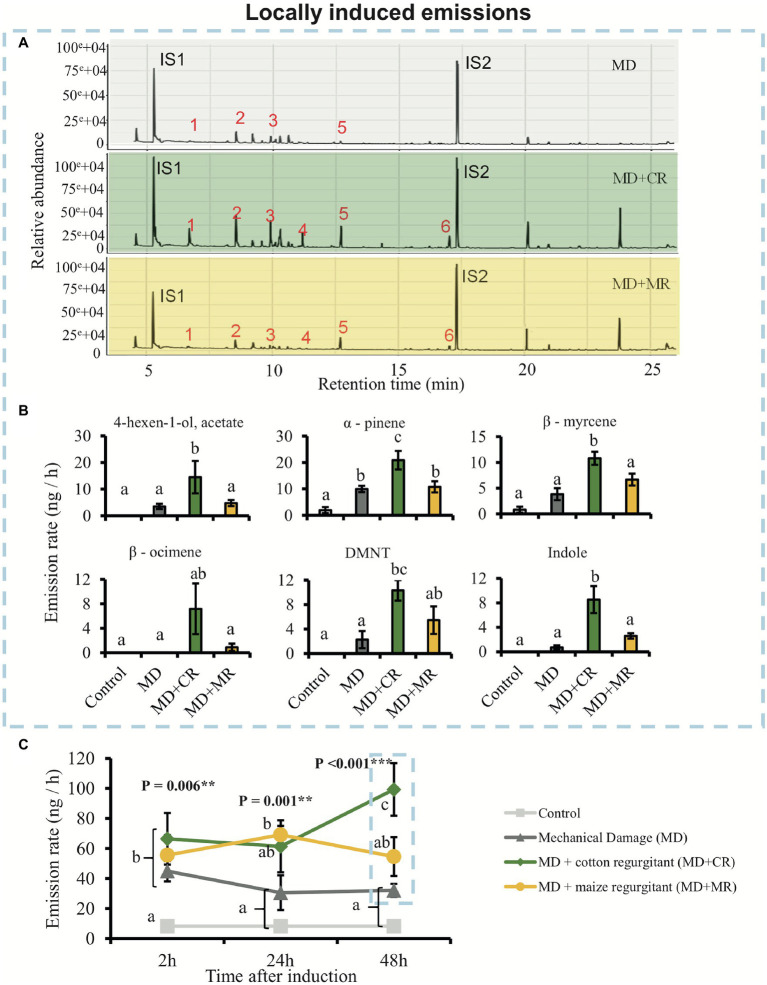
Cotton plants locally emit different amount of volatiles in response to elicitation by different caterpillar regurgitants. Plants were subjected to the following treatments: undamaged (Control), mechanically damaged (MD), mechanically damaged and application of regurgitant of cotton-fed *Spodoptera exigua* (MD + CR), and mechanically damaged and application of regurgitant of maize-fed *S. exigua* (MD + MR; *n* = 4–5). **(A)** Typical GC-MS chromatograms of HIPV from cotton plants 48 h after elicitation. The identities of the compounds are 1: 4-hexen-1-ol, acetate; 2: α-pinene; 3: β-myrcene; 4: β-ocimene; 5: DMNT; 6: Indole; and IS1 and IS2: internal standards (20 ng/μl), *n*-octane and nonyl acetate, respectively. **(B)** Average (±SE) of the most representative compounds emitted by cotton plants 48 h after elicitation. Different letters indicate significant differences between treatments (*p* < 0.05). **(C)** Average (±SE) of total amount of volatiles emitted by treated cotton plants 2, 24, and 48 h after elicitation. Different letters indicate significant differences between treatments within each time point. *p* values are given for treatments [generalized linear model (GLM; family, Gaussian)] followed by pairwise comparisons of Least Squares Means (LSMeans). ^**^p < 0.01, ^***^p < 0.001.

To evaluate the effect of HAMPs and DAMPs on the induction of plant volatile emissions, we measured the volatiles emitted by cotton plants treated with the regurgitant of *S. exigua* fed on either cotton or maize plants. Overall, plants treated with *S. exigua* regurgitants emitted more volatiles than the mechanically damaged plants and that untreated plants (Figure 2). We found differential effects depending on whether the regurgitant originated from maize-fed or from cotton-fed caterpillars. These effects were larger after 48 h of elicitation (Figures 2A,C). After 24 h, the plants responded similarly to the application of regurgitant of both cotton-fed and maize-fed caterpillars (Figure 2C). Interestingly, only after 48 h the emission rate of HIPVs in plants treated with the regurgitant of maize-fed caterpillars did not differ from the volatiles emitted by mechanically damaged plants (Figures 2A,C). On the other hand, plants treated with the regurgitant of cotton-fed caterpillars had a more pronounced response after 48 h (Figures 2A,C). During the first 24 h, a total of seven compounds were detected (Supplementary Tables 1, 2). Furthermore, (*E*)-4,8–dimethyl–1,3,7-nonatriene (DMNT), (*E,E*)-4,8,12-trimethyltrideca-1,3,7,11-tetraene (TMTT), and indole were emitted only after 48 h of elicitation (Figure 2B; Supplementary Tables 1–3). The most abundant compounds emitted from plants treated with the regurgitant of cotton-fed caterpillars were 4-hexen-1-ol, acetate; α-pinene; β-myrcene; β-ocimene; DMNT, and Indole (Figures 2A,B). Most of these compounds were emitted at higher concentrations by plants treated with the regurgitant of cotton-fed *S. exigua* than by plants treated with the regurgitant of maize-fed caterpillars.

**Figure 3:**
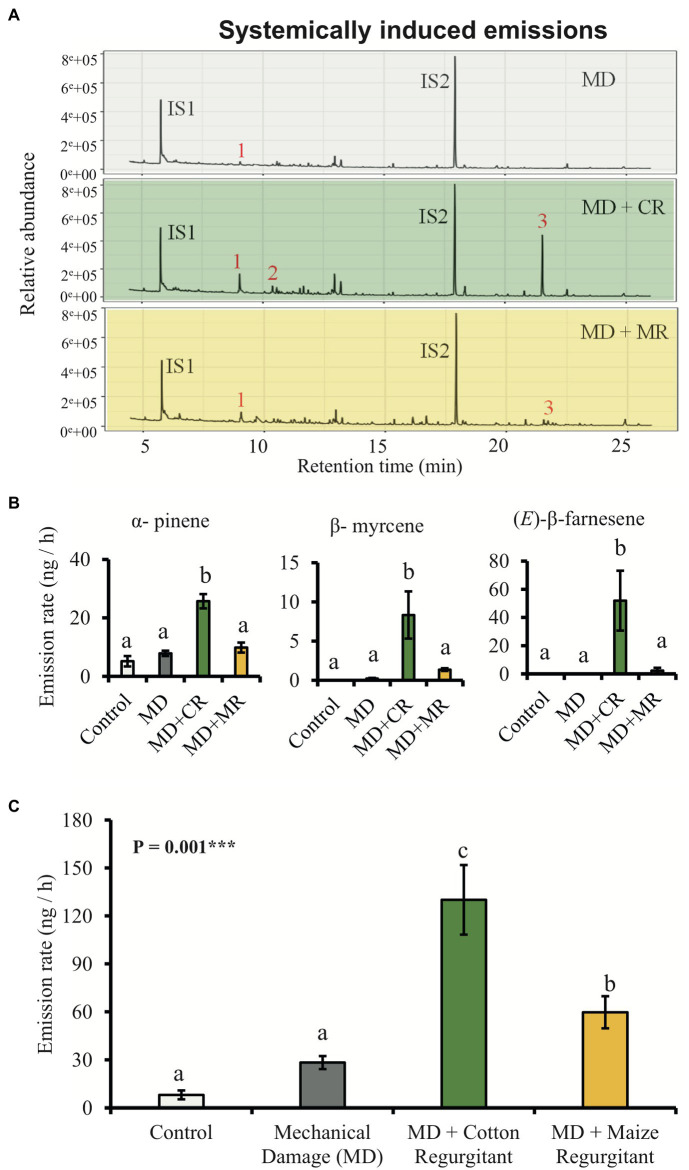
Cotton plants systemically emit different amount of volatiles in response to elicitation by different caterpillar regurgitants. Plants were subjected to the following treatments: undamaged (Control), mechanically damaged (MD), and application of regurgitant of cotton-fed *S. exigua* (MD + CR), and mechanically damaged and application of regurgitant of maize-fed *S. exigua* (MD + MR; *n* = 6). **(A)** Typical GC-MS chromatograms of HIPV from cotton plants 48 h after elicitation. The identities of the compounds are: 1: α-pinene, 2: β-myrcene, 3: (*E*)-β–farnesene, IS1 and IS2: internal standards (20 ng/μl), and *n*-octane and nonyl acetate, respectively. **(B)** Average (±SE) of the most representative compounds emitted by cotton plants 48 h after elicitation. **(C)** Average (±SE) of total amount of volatiles emitted by systemically treated cotton plants 48 h after elicitation. Different letters indicate significant differences among treatments (*p* < 0.05). *p* values are given for treatments [GLM (family, Gaussian)] followed by pairwise comparisons of LSMeans. ^***^p < 0.001 and ^*^p < 0.05.

**Figure 4:**
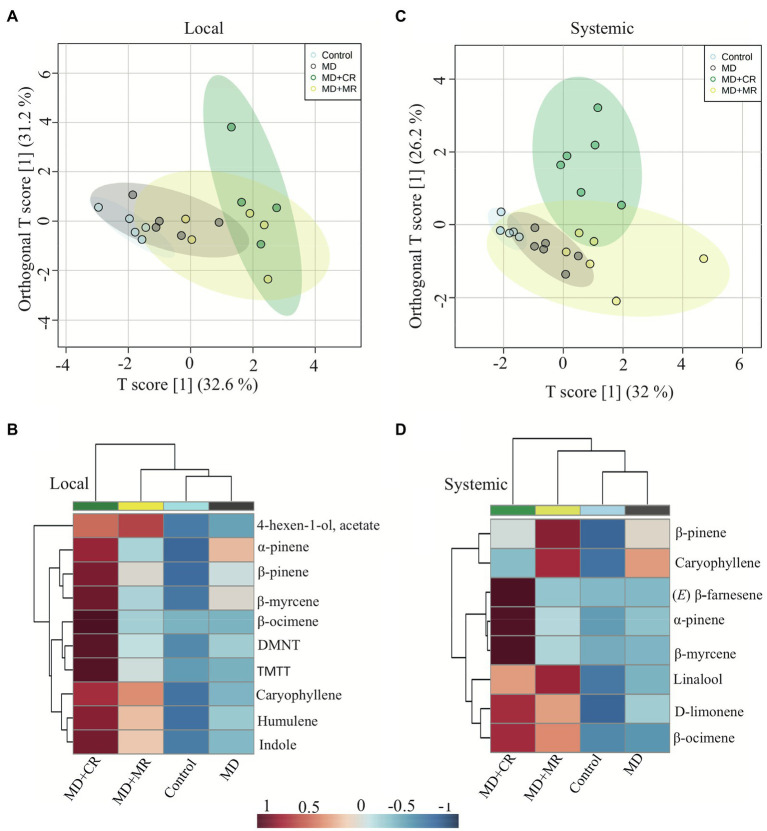
Differences in local and systemically emitted volatiles by cotton plants in response to elicitation by different caterpillar regurgitant. Plants were subjected to the following treatments: undamaged (Control), mechanically damaged (MD), and application of regurgitant of cotton-fed *S. exigua* (MD + CR), and mechanically damaged and application of regurgitant of maize-fed *S. exigua* (MD + MR; *n* = 4–6). The HIPVs were collected after 48 h of elicitation. **(A)** Results of a orthogonal partial least squares discriminant analysis (OPLS-DA) and **(B)** hierarchical clustering heatmaps of the local emission of volatiles by cotton plant treated with different regurgitants. **(C)** Results of a discriminant analysis (OPLS-DA) and **(D)** hierarchical clustering heatmaps of the systemically emitted volatiles by cotton after treatment with different regurgitants.

The HIPVs emitted by systemic leaves of cotton plants treated with the regurgitant of either cotton-fed or maize-fed *S. exigua* caterpillars were collected after 48 h. The emission rate of volatiles showed the same patterns as the patterns observed for locally induced plants (Figures 3A,C). In general, plants treated with the regurgitant of cotton-fed caterpillar emitted more volatiles than the plants treated with the regurgitant of maize-fed caterpillars. After 48 h of elicitation, we detected eight different volatiles (Supplementary Table 4). Three of them were significantly higher in plants treated with the regurgitant of cotton-fed caterpillars (Figures 3A,B). The monoterpene β-myrcene and the sesquiterpene (*E*)-β-farnesene were emitted mostly by plants treated with the regurgitant of cotton-fed caterpillars, whereas the monoterpene α-pinene were emitted by plants treated with both types of regurgitant, but in higher amounts by plants treated with the regurgitant of cotton-fed caterpillars (Figures 3A,B). Multivariate analyses, OPLS-DA and hierarchical clustering heatmaps, show clear differences in the volatile emission patters after the different plant elicitation treatments of local induction (Figures 4A,B) and systemic induction (Figures 4C,D).

**Figure 5:**
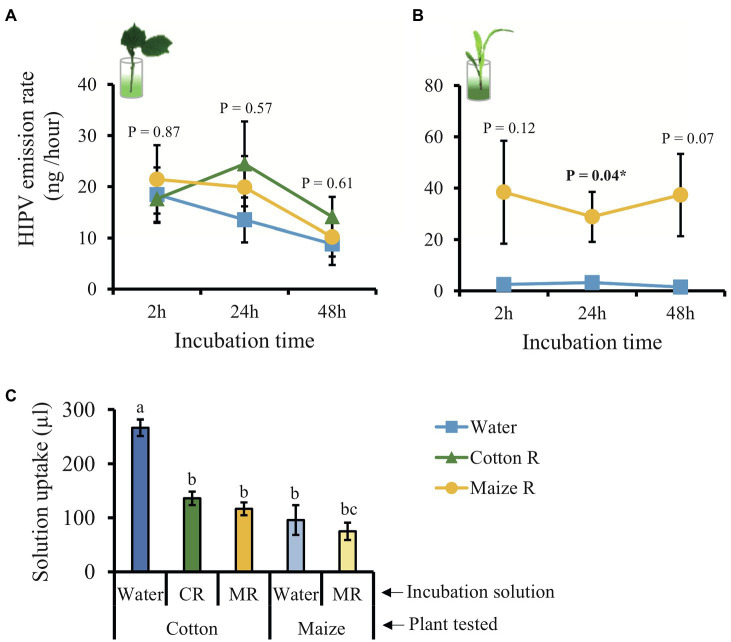
Incubation in caterpillar regurgitant does not induce volatile emissions in cotton plants. **(A)** Total emission of volatiles released by cotton plants incubated in water (blue line), regurgitant of cotton–fed *S. exigua* (green line) and regurgitant of maize–fed *S. exigua* (yellow line) after 2, 24, and 48 h (*n* = 4–6). **(B)** Total emission of volatiles released by maize plants (*n* = 4) incubated in water (blue line) and regurgitant from maize–fed *S. exigua* (yellow line) after 2, 24, and 48 h. **(C)** Mean quantity of solution taken up by either cotton or maize plants over 48 h. Lines and bars represent the average (±SE). Different letters indicate significant differences among treatments (*p* < 0.05). *p* values are given for treatments [GLM (family, Gaussian)] followed by pairwise comparisons of *LSMeans*. ^***^p < 0.001 and ^*^p < 0.05.

To evaluate the effect of HAMPs and minimizing the effect of mechanical damage on the induction of volatile emissions, we measured volatiles in plants that were incubated in a solution of caterpillar regurgitant using the method developed by Turlings et al. (2000). Unexpectedly, cotton plants incubated in a regurgitant solution did not show a pronounced induction of volatile emission (Figure 5A; Supplementary Figure 2). Nevertheless, the plants emitted three compounds independently of the treatment (α-pinene, β-myrcene, and caryophyllene) plus DMNT, which was emitted only by plants incubated in regurgitant of cotton-fed caterpillars (Supplementary Table 5). Maize plants were used as a positive control and showed the expected pattern of responses, especially after 24 h of incubation (Figure 5B; Supplementary Table 6; Supplementary Figure 3). The amounts of regurgitant solution uptaken by the plants did not differ, showing that this is not the reason why cotton plants did not respond to the treatments (Figure 5C).

### Volicitin Is Present in the Regurgitant of *Spodoptera exigua* Fed on Cotton Plants

**Figure 6:**
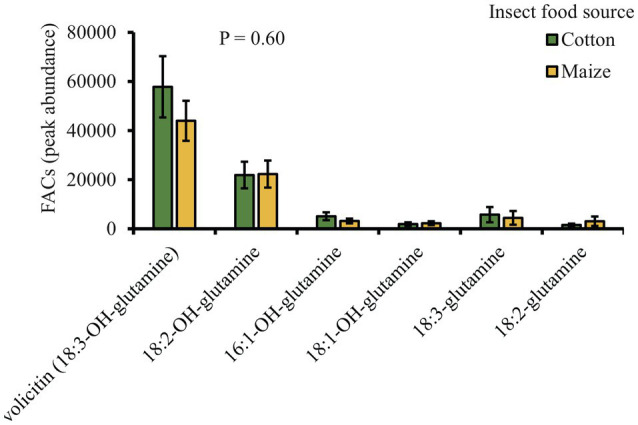
Volicitin and volicitin-like compounds are present in the regurgitant of *S. exigua* caterpillars fed on cotton and maize leaves (*n* = 5). Bars represent average (±SE). *p* values are given for treatment comparisons [GLM (family, Gaussian)], followed by pairwise comparisons of LSMeans.

The chemical analysis of *S. exigua* regurgitant fed on either cotton or maize plants revealed the presence of six different fatty acid amino acids conjugates (FACs): 18:3-OH-glutamine (volicitin), 18:2-OH-glutamine, 16:1-OH-glutamine, 18:01-OH-glutamine, 18:03-glutamine, and 18:2-glutamine. Interestingly, all the six FACs found in the regurgitant showed similar levels regardless of the plant food source that the insect fed on (Figure 6).

## Discussion

The identification of both DAMPs and HAMPs, and the evaluation of their relative contribution to triggering plant defenses are essential to understand the factors that regulate plant-herbivore interactions. It is known that wounding itself and/or elicitors present in the regurgitant of the herbivores can trigger the emission of plant volatiles (Mithöfer et al., 2005; Heil and Karban, 2010; Turlings and Erb, 2018). The specificity of the response depends on the recognition of these molecules by the plant and the responses may be different for different plant species. Our study shows that volicitin, a potent HAMP, is present in the regurgitant of both cotton-fed and maize-fed *S. exigua* caterpillars, but only the regurgitant from cotton-fed caterpillars elicit a clear induction of volatiles in cotton plants. Therefore, it is unlikely that volicitin or any other FAC play a role in the induction, but that other, yet unidentified, DAMPs are involved in the regulation of HIPV responses in cotton plants.

Our study is in good agreement with previous studies that show that mechanical damage and the application of *S. exigua* regurgitant on cotton leaves induces higher rates of overall responses and systemic volatile emissions than only mechanical damage (McCall et al., 1994; Paré and Tumlinson, 1997; Röse and Tumlinson, 2005). Our finding that the response elicited by regurgitant of cotton-fed *S. exigua* is stronger than the response elicited by the regurgitant of maize-fed *S. exigua* suggests the involvement of specific cotton-derived DAMPs. The self-recognition by cotton plants when treated with regurgitant of *S. exigua* fed on cotton leaves might be responsible for the observed induction of HIPVs emission (Huffaker et al., 2006; Yamaguchi et al., 2006; Heil and Land, 2014; Duran-Flores and Heil, 2016). This can only be confirmed when, the chemical nature of the responsible molecules are known. Few molecules have been identified as DAMPs, e.g., extracellular ATP, extracellular DNA, and extracellular sucrose. These molecules may also include cell-wall fragments (e.g., oligosaccharides, oligogalacturonides; Bonaventure et al., 2011; Duran-Flores and Heil, 2014). Turlings et al. (1993) found that incubating maize plants in a maize leaf juice solution induced a weak, but significant emission of volatiles, which did not occur when the plants were incubated only with water. Yet, incubating the plants in caterpillar regurgitant was far more potent in inducing volatile emissions (Turlings et al., 1993), implying that HAMPs play a more important role than DAMPs in the maize-caterpillar interaction. Mattiacci et al. (1994, 1995) found the same for the volatile responses of cabbage plants to the regurgitant of *Pieris* caterpillars. For bean plants, the application of leaf homogenate is sufficient to induce the production of reactive oxygen species and extra floral nectar (Duran-Flores and Heil, 2014), but they are also responsive to a HAMP, a peptide which has been named inceptin (Schmelz et al., 2006, 2009). Some authors have argued that wounding and the resulting exposure to DAMPs is enough to trigger the emission of HIPVs (Mithöfer et al., 2005; Heil and Karban, 2010), but the relative contribution of DAMPs remains unclear. Most likely, DAMP and HAMP molecules are used in combination by plants to identify the nature of the organisms that initiate an interaction with them reviewed in Dicke (2016).

It is evident that chemical cues in herbivore oral secretions play key roles in eliciting plants defense, and the outcome of the response can vary among herbivores species (Acevedo et al., 2015; Schmelz, 2015; Turlings and Erb, 2018). Several elicitors have been identified, e.g., volicitin, caeliferins, inceptins, and β-glucosidase (Mattiacci et al., 1995; Alborn et al., 1997; Schmelz et al., 2006). FACs like volicitin are oral secretion components of many lepidopteran, but also present in other insect’s order such as Orthoptera and Diptera (Yoshinaga et al., 2007, 2010). They are sufficient to elicit herbivory-specific responses in several plant species including maize and the wild-type tobacco (Turlings et al., 1993; Alborn et al., 1997; Halitschke et al., 2001). Our results showed that volicitin levels in the regurgitant of both cotton- and maize-fed *S. exigua* are the same. The fact that the regurgitant of cotton-fed caterpillars induced stronger HIPVs emissions than the regurgitant of maize-fed caterpillars, strongly suggests that other types of elicitors are involved. Indeed, it has been suggested that insect oral secretion may contain more than a single elicitor with eliciting activity and that these can act synergically or independently to regulate plant defense responses (Spiteller et al., 2001; Acevedo et al., 2017; Basu et al., 2018; Jones et al., 2019). FACs are expected to act specifically and, therefore, different plant species recognize them by different mechanisms, or they are active only in certain plants where they induce a *de novo* production of JA e.g., tobacco, eggplant, maize, and soybean (Halitschke et al., 2001; von Dahl et al., 2007; Wu et al., 2007; Schmelz et al., 2009). Spiteller et al. (2001) found that synthetic volicitin does not induced volatiles in lima bean and cotton, but the FAC *N*-lino-lenoylglutamine does induced the biosynthesis of volatiles in lima bean (Koch et al., 1999). Also, FACs do not affect JA production in *Arabidopsis* (Schmelz et al., 2009). The species-specific responses are likely due to plant-specific receptors (pattern recognition *receptor*, PRRs). One such receptor was recently identified inceptin (Steinbrenner et al., 2020), confirming that they play a key role in elicitor perception (Truitt et al., 2004; Santamaria et al., 2018; Malik et al., 2020). To date, this research area still lacks enough knowledge to fully characterize specific HAMPs-PRRs interactions.

The overall blend of HIPV emitted by cotton plants treated with *S. exigua* regurgitant was composed mainly by monoterpenes and sesquiterpenes (Figures 2, 4). These results are in a good agreement with those found by McAuslane et al. (1997); Röse and Tumlinson (2005) and Loughrin et al. (1995). The main constitutive terpenoids emitted by treated plants were: α-pinene, β-pinene, myrcene, and caryophyllene. They are known to be stored in the glands located near the surface of cotton leaves and be immediately released when the glands are ruptured (Elzen et al., 1985; Rose et al., 1996). Previous studies have shown that in addition to these constitutive volatiles, attack by *S. exigua* caterpillars, also results in *de novo* biosynthesis of several other volatiles that are released with considerable delay (Loughrin et al., 1994; McCall et al., 1994). In accordance, 48 h after treatment, we observed an additional release of mainly non-cyclic terpenoids, which included, (*E*)-β-ocimene, linalool, (*E*)-4,8-dimethyl-1,3,7-nonatriene (DMNT), and (*E*)-β-farnesene. These truly inducible volatiles are also systemically released from non-attacked leaves of cotton plants (Paré and Tumlinson, 1998; Röse and Tumlinson, 2005). The same pattern of emission was found in our study, but we did not detect the presence of DMNT, (*E,E*)-4,8,12-trimethyl-1,3,7,11-tridecatetraene (TMTT), and indole in the systemic response. Indeed, Rose et al. (1996, 2006) detected the systemic release of these compounds only after 3 or 4 days. We collected the HIPVs after 48 h damage, which might have been too early to find these compounds in the systemic leaves in our experiment. Somewhat surprisingly, incubating cotton plants in *S. exigua* regurgitant solution did not result in any induction of HIPV. This is in sharp contrast to maize plants that respond strongly to the incubation in regurgitant solution of *S. exigua* with the emission of typical maize HIPVs (Turlings et al., 1993), as we confirm here (Figure 5). Importantly, the fact that the cotton plants treated with regurgitant from cotton-fed caterpillars emitted larger quantities of volatiles than plants treated with regurgitant from maize-fed caterpillars, is in line with the notion that DAMPs are also involved in the response (Duran-Flores and Heil, 2016). Testing additional types of regurgitant and conducting similar experiments with other plant species may shed more light on the importance of self-damage recognition. It appears that this will vary for different plant species, as, for instance, maize plants respond weaker to cotton-derived regurgitant than to maize-derived regurgitant but stronger to soybean-derived regurgitant (Turlings et al., 1993).

We draw two primary conclusions from our work investigating the factors involved in caterpillar induced cotton volatiles. Firstly, mechanical damage alone is not sufficient to induce a full response and elicitors present in caterpillar oral secretions enhance the response. Secondly, the difference in emissions from plants treated with cotton- and maize-derived secretions may imply that unidentified cotton DAMPs, help the plants to recognize self-damage. Cotton plants have been shown to particularly amenable to priming with inducible volatiles; they can become considerably resistant to insect attack if they have been exposed to volatiles from attacked plants (Renou et al., 2011; Llandres et al., 2018). Therefore, further unraveling the respective roles of HAMPs and DAMPs present in the oral secretion of caterpillars fed in inducing cotton leaf volatiles may provide crucial information that can help to improve cotton defenses against important pests in an agricultural context.

## Data Availability Statement

The raw data supporting the conclusions of this article will be made available by the authors, without undue reservation.

## Author Contributions

TT and CA designed the study. CA and GB collected data and analyzed and interpreted the data. GG developed the method to analyze volicitin. CA and GA wrote the first draft of the manuscript. All authors contributed to the article and approved the submitted version.

## Conflict of Interest

The authors declare that the research was conducted in the absence of any commercial or financial relationship that could be construed as a potential conflict of interest.

## Publisher’s Note

All claims expressed in this article are solely those of the authors and do not necessarily represent those of their affiliated organizations, or those of the publisher, the editors and the reviewers. Any product that may be evaluated in this article, or claim that may be made by its manufacturer, is not guaranteed or endorsed by the publisher.

## References

[ref1] Acevedo F. E. Peiffer M. Tan C. W. Stanley B. A. Stanley A. Wang J.. (2017). Fall armyworm-associated gut bacteria modulate plant defense responses. Mol. Plant-Microbe Interact. 30, 127–137. 10.1094/MPMI-11-16-0240-R, PMID: 28027025

[ref2] Acevedo F. E. Rivera-Vega L. J. Chung S. H. Ray S. Felton G. W. (2015). Cues from chewing insects—the intersection of DAMPs, HAMPs, MAMPs and effectors. Curr. Opin. Plant Biol. 26, 80–86. 10.1016/j.pbi.2015.05.029, PMID: 26123394

[ref3] Alborn H. T. Hansen T. V. Jones T. H. Bennett D. C. Tumlinson J. H. Schmelz E. A.. (2007). Disulfooxy fatty acids from the American bird grasshopper *Schistocerca americana*, elicitors of plant volatiles. Proc. Natl. Acad. Sci. U. S. A. 104, 12976–12981. 10.1073/pnas.0705947104, PMID: 17664416 PMC1941812

[ref4] Alborn H. T. Jones T. H. Stenhagen G. S. Tumlinson J. H. (2000). Identification and synthesis of volicitin and related components from beet armyworm oral secretions. J. Chem. Ecol. 26, 203–220. 10.1023/A:1005401814122

[ref5] Alborn H. T. Turlings T. C. J. Jones T. H. Stenhagen G. Loughrin J. H. Tumlinson J. H. (1997). An elicitor of plant volatiles from beet armyworm oral secretion. Science 276, 945–949. 10.1126/science.276.5314.945

[ref6] Aljbory Z. Chen M. S. (2018). Indirect plant defense against insect herbivores: a review. Insect Sci. 25, 2–23. 10.1111/1744-7917.12436, PMID: 28035791

[ref7] Basu S. Varsani S. Louis J. (2018). Altering plant defenses: herbivore-associated molecular patterns and effector arsenal of chewing herbivores. Mol. Plant Microbe Interact. 31, 13–21. 10.1094/MPMI-07-17-0183-FI, PMID: 28840787

[ref8] Bonaventure G. VanDoorn A. Baldwin I. T. (2011). Herbivore-associated elicitors: FAC signaling and metabolism. Trends Plant Sci. 16, 294–299. 10.1016/j.tplants.2011.01.006, PMID: 21354852

[ref9] Choi H. W. Klessig D. F. (2016). DAMPs, MAMPs, and NAMPs in plant innate immunity. BMC Plant Biol. 16:232. 10.1186/s12870-016-0921-2, PMID: 27782807 PMC5080799

[ref10] Chong J. Wishart D. S. Xia J. (2019). Using metaboanalyst 4.0 for comprehensive and integrative metabolomics data analysis. Curr. Protoc. Bioinformatics 68:e86. 10.1002/cpbi.86, PMID: 31756036

[ref11] De Lange E. S. Laplanche D. Guo H. Xu W. Vlimant M. Erb M.. (2020). *Spodoptera frugiperda* caterpillars suppress herbivore-induced volatile emissions in maize. J. Chem. Ecol. 46, 344–360. 10.1007/s10886-020-01153-x, PMID: 32002720

[ref12] Devaiah S. P. Mahadevappa G. H. Shetty H. S. (2009). Induction of systemic resistance in pearl millet (*Pennisetum glaucum*) against downy mildew (*Sclerospora graminicola*) by *Datura metel* extract. Crop Prot. 28, 783–791. 10.1016/j.cropro.2009.04.009

[ref13] Dicke M. (2016). Plant phenotypic plasticity in the phytobiome: a volatile issue. Curr. Opin. Plant Biol. 32, 17–23. 10.1016/j.pbi.2016.05.004, PMID: 27267277

[ref14] Dicke M. Baldwin I. T. (2010). The evolutionary context for herbivore-induced plant volatiles: beyond the ‘cry for help’. Trends Plant Sci. 15, 167–175. 10.1016/j.tplants.2009.12.002, PMID: 20047849

[ref15] Dicke M. Sabelis M. W. de Jong M. (1988). Analysis of prey preference in phytoseiid mites by using an olfactometer, predation models and electrophoresis. Exp. Appl. Acarol. 5, 225–241. 10.1007/BF02366096

[ref16] Duran-Flores D. Heil M. (2014). Damaged-self recognition in common bean (*Phaseolus vulgaris*) shows taxonomic specificity and triggers signaling via reactive oxygen species (ROS). Front. Plant Sci. 5:585. 10.3389/fpls.2014.00585, PMID: 25400650 PMC4215620

[ref17] Duran-Flores D. Heil M. (2016). Sources of specificity in plant damaged-self recognition. Curr. Opin. Plant Biol. 32, 77–87. 10.1016/j.pbi.2016.06.019, PMID: 27421107

[ref18] Elzen G. W. Williams H. J. Bell A. A. Stipanovic R. D. Vinson S. B. (1985). Quantification of volatile terpenes of glanded and glandless *Gossypium hirsutum* L. cultivars and lines by gas chromatography. J. Agric. Food Chem. 33, 1079–1082. 10.1021/jf00066a015

[ref19] Erb M. Reymond P. (2019). Molecular interactions between plants and insect herbivores. Annu. Rev. Plant Biol. 70, 527–557. 10.1146/annurev-arplant-050718-095910, PMID: 30786233

[ref20] Erb M. Veyrat N. Robert C. A. Xu H. Frey M. Ton J.. (2015). Indole is an essential herbivore-induced volatile priming signal in maize. Nat. Commun. 6:6273. 10.1038/ncomms7273, PMID: 25683900 PMC4339915

[ref21] Eveleens K. G. Van Den Bosch R. Ehler L. E. (1973). Secondary outbreak induction of beet armyworm by experimental insecticide applications in cotton in California. Environ. Entomol. 2, 497–504. 10.1093/ee/2.4.497

[ref22] Farmer E. E. (2014). Leaf Defence. Oxford, UK: Oxford Univ. Press.

[ref23] Felton G. W. Tumlinson J. H. (2008). Plant–insect dialogs: complex interactions at the plant–insect interface. Curr. Opin. Plant Biol. 11, 457–463. 10.1016/j.pbi.2008.07.001, PMID: 18657469

[ref24] Fürstenberg-Hägg J. Zagrobelny M. Bak S. (2013). Plant defense against insect herbivores. Int. J. Mol. Sci. 14, 10242–10297. 10.3390/ijms140510242, PMID: 23681010 PMC3676838

[ref25] Green T. R. Ryan C. A. (1972). Wound-induced proteinase inhibitor in plant leaves: a possible defense mechanism against insects. Science 175, 776–777. 10.1126/science.175.4023.776, PMID: 17836138

[ref26] Greenberg S. Sappington T. Legaspi B. Liu T.-X. Setamou M. (2001). Feeding and life history of *Spodoptera exigua* (Lepidoptera: Noctuidae) on different host plants. Ann. Entomol. Soc. Am. 94, 566–575. 10.1603/0013-8746(2001)094[0566:FALHOS]2.0.CO;2

[ref27] Gust A. A. Pruitt R. Nürnberger T. (2017). Sensing danger: key to activating plant immunity. Trends Plant Sci. 22, 779–791. 10.1016/j.tplants.2017.07.005, PMID: 28779900

[ref28] Hagenbucher S. Olson D. M. Ruberson J. R. Wäckers F. L. Romeis J. (2013). Resistance mechanisms against arthropod herbivores in cotton and their interactions with natural enemies. Crit. Rev. Plant Sci. 32, 458–482. 10.1080/07352689.2013.809293

[ref29] Halitschke R. Schittko U. Pohnert G. Boland W. Baldwin I. T. (2001). Molecular interactions between the specialist herbivore *Manduca sexta* (Lepidoptera, Sphingidae) and its natural host *Nicotiana attenuata*. III. Fatty acid-amino acid conjugates in herbivore oral secretions are necessary and sufficient for herbivore-specific plant responses. Plant Physiol. 125, 711–717. 10.1104/pp.125.2.711, PMID: 11161028 PMC64872

[ref30] Heil M. (2009). Damaged-self recognition in plant herbivore defence. Trends Plant Sci. 14, 356–363. 10.1016/j.tplants.2009.04.002, PMID: 19540148

[ref31] Heil M. Karban R. (2010). Explaining evolution of plant communication by airborne signals. Trends Ecol. Evol. 25, 137–144. 10.1016/j.tree.2009.09.010, PMID: 19837476

[ref32] Heil M. Land W. G. (2014). Danger signals–damaged-self recognition across the tree of life. Front. Plant Sci. 5:578. 10.3389/fpls.2014.00578, PMID: 25400647 PMC4215617

[ref33] Henry G. Thonart P. Ongena M. (2012). PAMPs, MAMPs, DAMPs and others: an update on the diversity of plant immunity elicitors. BASE 16, 257–268.

[ref34] Hou S. Liu Z. Shen H. Wu D. (2019). Damage-associated molecular pattern-triggered immunity in plants. Front. Plant Sci. 10:646. 10.3389/fpls.2019.00646, PMID: 31191574 PMC6547358

[ref35] Huffaker A. Pearce G. Ryan C. A. (2006). An endogenous peptide signal in *Arabidopsis* activates components of the innate immune response. Proc. Natl. Acad. Sci. U. S. A. 103, 10098–10103. 10.1073/pnas.0603727103, PMID: 16785434 PMC1502512

[ref36] Huffaker A. Pearce G. Veyrat N. Erb M. Turlings T. C. J. Sartor R.. (2013). Plant elicitor peptides are conserved signals regulating direct and indirect antiherbivore defense. Proc. Natl. Acad. Sci. U. S. A. 110, 5707–5712. 10.1073/pnas.1214668110, PMID: 23509266 PMC3619339

[ref37] Huffaker A. Ryan C. A. (2007). Endogenous peptide defense signals in *Arabidopsis* differentially amplify signaling for the innate immune response. Proc. Natl. Acad. Sci. U. S. A. 104, 10732–10736. 10.1073/pnas.0703343104, PMID: 17566109 PMC1965581

[ref38] Jones A. G. Mason C. J. Felton G. W. Hoover K. (2019). Host plant and population source drive diversity of microbial gut communities in two polyphagous insects. Sci. Rep. 9:2792. 10.1038/s41598-019-39163-9, PMID: 30808905 PMC6391413

[ref39] Karban R. Baldwin I. T. (1997). Induced Responses to Herbivory. Chicago: University of Chicago Press.

[ref40] Koch T. Krumm T. Jung V. Engelberth J. Boland W. (1999). Differential induction of plant volatile biosynthesis in the lima bean by early and late intermediates of the octadecanoid-signaling pathway. Plant Physiol. 121, 153–162. 10.1104/pp.121.1.153, PMID: 10482670 PMC59363

[ref41] Llandres A. L. Almohamad R. Brévault T. Renou A. Téréta I. Jean J.. (2018). Plant training for induced defense against insect pests: a promising tool for integrated pest management in cotton. Pest Manag. Sci. 74, 2004–2012. 10.1002/ps.5039, PMID: 29667361

[ref42] Lotze M. T. Zeh H. J. Rubartelli A. Sparvero L. J. Amoscato A. A. Washburn N. R.. (2007). The grateful dead: damage-associated molecular pattern molecules and reduction/oxidation regulate immunity. Immunol. Rev. 220, 60–81. 10.1111/j.1600-065X.2007.00579.x, PMID: 17979840

[ref43] Loughrin J. H. Manukian A. Heath R. R. Tumlinson J. H. (1995). Volatiles emitted by different cotton varieties damaged by feeding beet armyworm larvae. J. Chem. Ecol. 21, 1217–1227. 10.1007/BF02228321, PMID: 24234527

[ref44] Loughrin J. H. Manukian A. Heath R. R. Turlings T. C. Tumlinson J. H. (1994). Diurnal cycle of emission of induced volatile terpenoids by herbivore-injured cotton plant. Proc. Natl. Acad. Sci. 91, 11836–11840. 10.1073/pnas.91.25.11836, PMID: 11607499 PMC45330

[ref45] Louis J. Peiffer M. Ray S. Luthe D. S. Felton G. W. (2013). Host-specific salivary elicitor(s) of European corn borer induce defenses in tomato and maize. New Phytol. 199, 66–73. 10.1111/nph.12308, PMID: 23627593

[ref46] Malik N. A. A. Kumar I. S. Nadarajah K. (2020). Elicitor and receptor molecules: orchestrators of plant defense and immunity. Int. J. Mol. Sci. 21:963. 10.3390/ijms21030963, PMID: 32024003 PMC7037962

[ref47] Mattiacci L. Dicke M. Posthumus M. A. (1994). Induction of parasitoid attracting synomone in *Brussels sprouts* plants by feeding of *Pieris brassicae* larvae: role of mechanical damage and herbivore elicitor. J. Chem. Ecol. 20, 2229–2247. 10.1007/BF02033199, PMID: 24242803

[ref48] Mattiacci L. Dicke M. Posthumus M. A. (1995). Beta-glucosidase: an elicitor of herbivore-induced plant odor that attracts host-searching parasitic wasps. Proc. Natl. Acad. Sci. U. S. A. 92, 2036–2040. 10.1073/pnas.92.6.2036, PMID: 11607516 PMC42418

[ref49] McAuslane H. J. Alborn H. T. Toth J. P. (1997). Systemic induction of terpenoid aldehydes in cotton pigment glands by feeding of larval *Spodoptera exigua*. J. Chem. Ecol. 23, 2861–2879. 10.1023/A:1022575313325

[ref50] McCall P. J. Turlings T. C. J. Loughrin J. Proveaux A. T. Tumlinson J. H. (1994). Herbivore-induced volatile emissions from cotton (*Gossypium hirsutum* L.) seedlings. J. Chem. Ecol. 20, 3039–3050. 10.1007/BF02033709, PMID: 24241975

[ref51] Mithöfer A. Wanner G. Boland W. (2005). Effects of feeding Spodoptera littoralis on lima bean leaves. II. Continuous mechanical wounding resembling insect feeding is sufficient to elicit herbivory-related volatile emission. Plant Physiol. 137, 1160–1168. 10.1104/pp.104.054460, PMID: 15728342 PMC1065415

[ref52] Naranjo S. E. Ruberson J. R. Sharma H. C. Wilson L. Wu K. (2008). “The present and future role of insect-resistant genetically modified cotton in IPM,” in Integration of Insect-Resistant Genetically Modified Crops Within IPM Programs. eds. Romeis J. Shelton A. M. Kennedy G. G. (Dordrecht: Springer), 159–194.

[ref53] Paré P. W. Tumlinson J. H. (1997). De novo biosynthesis of volatiles induced by insect herbivory in cotton plants. Plant Physiol. 114, 1161–1167. 10.1104/pp.114.4.1161, PMID: 12223763 PMC158408

[ref54] Paré P. W. Tumlinson J. H. (1998). Cotton volatiles synthesized and released distal to the site of insect damage. Phytochemistry 47, 521–526. 10.1016/S0031-9422(97)00442-1

[ref55] Paré P. W. Tumlinson J. H. (1999). Plant volatiles as a defense against insect herbivores. Plant Physiol. 121, 325–332. 10.1104/pp.121.2.325, PMID: 10517823 PMC1539229

[ref56] Pearce G. Ryan C. A. (2003). Systemic signaling in tomato plants for defense against herbivores: isolation and characterization of three novel defense-signaling glycopeptide hormones coded in a single precursor gene. J. Biol. Chem. 278, 30044–30050. 10.1074/jbc.M304159200, PMID: 12748180

[ref57] Pearce G. Strydom D. Johnson S. Ryan C. A. (1991). A polypeptide from tomato leaves induces wound-inducible proteinase inhibitor proteins. Science 253, 895–897. 10.1126/science.253.5022.895, PMID: 17751827

[ref58] Quintana-Rodriguez E. Duran-Flores D. Heil M. Camacho-Coronel X. (2018). Damage-associated molecular patterns (DAMPs) as future plant vaccines that protect crops from pests. Sci. Hortic. 237, 207–220. 10.1016/j.scienta.2018.03.026

[ref59] Renou A. Téréta I. Togola M. (2011). Manual topping decreases bollworm infestations in cotton cultivation in Mali. Crop Prot. 30, 1370–1375. 10.1016/j.cropro.2011.05.020

[ref60] Rose U. S. R. Lewis J. Tumlinson J. H. (2006). Extrafloral nectar from cotton (*Gossypium hirsutum*) as a food source for parasitic wasps. Funct. Ecol. 20, 67–74. 10.1111/j.1365-2435.2006.01071.x

[ref61] Rose U. S. Manukian A. Heath R. R. Tumlinson J. H. (1996). Volatile semiochemicals released from undamaged cotton leaves (a systemic response of living plants to caterpillar damage). Plant Physiol. 111, 487–495. 10.1104/pp.111.2.487, PMID: 12226304 PMC157859

[ref62] Röse U. S. Tumlinson J. H. (2004). Volatiles released from cotton plants in response to *Helicoverpa zea* feeding damage on cotton flower buds. Planta 218, 824–832. 10.1007/s00425-003-1162-9, PMID: 14625774

[ref63] Röse U. S. Tumlinson J. H. (2005). Systemic induction of volatile release in cotton: how specific is the signal to herbivory? Planta 222, 327–335. 10.1007/s00425-005-1528-2, PMID: 15856281

[ref64] Santamaria M. E. Arnaiz A. Gonzalez-Melendi P. Martinez M. Diaz I. (2018). Plant perception and short-term responses to phytophagous insects and mites. Int. J. Mol. Sci. 19:1356. 10.3390/ijms19051356, PMID: 29751577 PMC5983831

[ref65] Schmelz E. A. (2015). Impacts of insect oral secretions on defoliation-induced plant defense. Curr. Opin. Insect Sci. 9, 7–15. 10.1016/j.cois.2015.04.002, PMID: 32846712

[ref66] Schmelz E. A. Carroll M. J. LeClere S. Phipps S. M. Meredith J. Chourey P. S.. (2006). Fragments of ATP synthase mediate plant perception of insect attack. Proc. Natl. Acad. Sci. U. S. A. 103, 8894–8899. 10.1073/pnas.0602328103, PMID: 16720701 PMC1482674

[ref67] Schmelz E. A. Engelberth J. Alborn H. T. Tumlinson J. H. Teal P. E. A. (2009). Phytohormone-based activity mapping of insect herbivore-produced elicitors. Proc. Natl. Acad. Sci. U. S. A. 106, 653–657. 10.1073/pnas.0811861106, PMID: 19124770 PMC2626758

[ref68] Spiteller D. Pohnert G. Boland W. (2001). Absolute configuration of volicitin, an elicitor of plant volatile biosynthesis from lepidopteran larvae. Tetrahedron Lett. 42, 1483–1485. 10.1016/S0040-4039(00)02290-5

[ref69] Steinbrenner A. D. Muñoz-Amatriaín M. Chaparro A. F. Aguilar-Venegas J. M. Lo S. Okuda S.. (2020). A receptor-like protein mediates plant immune responses to herbivore-associated molecular patterns. Proc. Natl. Acad. Sci. U. S. A. 117, 31510–31518. 10.1073/pnas.2018415117, PMID: 33229576 PMC7733821

[ref001] Team R. C. (2001). “R installation and administration”. R Foundation for Statistical Computing, Vienna, Austria, 2015a., PMID: 32846712

[ref70] Truitt C. L. Wei H. X. Pare P. W. (2004). A plasma membrane protein from *Zea mays* binds with the herbivore elicitor volicitin. Plant Cell 16, 523–532. 10.1105/tpc.017723, PMID: 14729912 PMC341921

[ref71] Turlings T. C. J. Alborn H. T. Loughrin J. H. Tumlinson J. H. (2000). Volicitin, an elicitor of maize volatiles in oral secretion of *Spodoptera exigua*: isolation and bioactivity. J. Chem. Ecol. 26, 189–202. 10.1023/A:1005449730052

[ref002] Turlings T. C. J. Davison A. C. TamÒ C. (2004). A six‐arm olfactometer permitting simultaneous observation of insect attraction and odour trapping. Physiol. Entomol. 29, 45–55. 10.1111/j.1365-3032.2004.0362.x

[ref72] Turlings T. C. J. Erb M. (2018). Tritrophic interactions mediated by herbivore-induced plant volatiles: mechanisms, ecological relevance, and application potential. Annu. Rev. Entomol. 63, 433–452. 10.1146/annurev-ento-020117-043507, PMID: 29324043

[ref73] Turlings T. C. J. Lengwiler U. B. Bernasconi M. L. Wechsler D. (1998). Timing of induced volatile emissions in maize seedlings. Planta 207, 146–152. 10.1007/s004250050466

[ref74] Turlings T. C. J. McCall P. J. Alborn H. T. Tumlinson J. H. (1993). An elicitor in caterpillar oral secretions that induces corn seedlings to emit chemical signals attractive to parasitic wasps. J. Chem. Ecol. 19, 411–425. 10.1007/BF00994314, PMID: 24248945

[ref75] Turlings T. C. J. Tumlinson J. H. Lewis W. J. (1990). Exploitation of herbivore-induced plant odors by host-seeking parasitic wasps. Science 250, 1251–1253. 10.1126/science.250.4985.1251, PMID: 17829213

[ref76] Turlings T. C. J. Wäckers F. (2004). “Recruitment of predators and parasitoids by herbivore-injured plants,” in Advances in Insect Chemical Ecology. eds. Cardé R. T. Millar J. G. (Cambridge: Cambridge University Press), 21–71.

[ref77] von Dahl C. C. Winz R. A. Halitschke R. Kühnemann F. Gase K. Baldwin I. T. (2007). Tuning the herbivore-induced ethylene burst: the role of transcript accumulation and ethylene perception in *Nicotiana attenuata*. Plant J. 51, 293–307. 10.1111/j.1365-313X.2007.03142.x, PMID: 17559506

[ref78] Wiklund S. Johansson E. Sjöström L. Mellerowicz E. J. Edlund U. Shockcor J. P.. (2008). Visualization of GC/TOF-MS-based metabolomics data for identification of biochemically interesting compounds using OPLS class models. Anal. Chem. 80, 115–122. 10.1021/ac0713510, PMID: 18027910

[ref79] Wu J. Baldwin I. T. (2009). Herbivory-induced signalling in plants: perception and action. Plant Cell Environ. 32, 1161–1174. 10.1111/j.1365-3040.2009.01943.x, PMID: 19183291

[ref80] Wu J. Hettenhausen C. Meldau S. Baldwin I. T. (2007). Herbivory rapidly activates MAPK signaling in attacked and unattacked leaf regions but not between leaves of *Nicotiana attenuata*. Plant Cell 19, 1096–1122. 10.1105/tpc.106.049353, PMID: 17400894 PMC1867352

[ref81] Yamaguchi Y. Pearce G. Ryan C. A. (2006). The cell surface leucine-rich repeat receptor for AtPep1, an endogenous peptide elicitor in *Arabidopsis*, is functional in transgenic tobacco cells. Proc. Natl. Acad. Sci. U. S. A. 103, 10104–10109. 10.1073/pnas.0603729103, PMID: 16785433 PMC1502513

[ref82] Yoshinaga N. Aboshi T. Ishikawa C. Fukui M. Shimoda M. Nishida R.. (2007). Fatty acid amides, previously identified in caterpillars, found in the cricket *Teleogryllus taiwanemma* and fruit fly *Drosophila melanogaster* larvae. J. Chem. Ecol. 33, 1376–1381. 10.1007/s10886-007-9321-2, PMID: 17566833

[ref83] Yoshinaga N. Alborn H. T. Nakanishi T. Suckling D. M. Nishida R. Tumlinson J. H.. (2010). Fatty acid-amino acid conjugates diversification in lepidopteran caterpillars. J. Chem. Ecol. 36, 319–325. 10.1007/s10886-010-9764-8, PMID: 20195891

[ref84] Zheng X. L. Cong X. P. Wang X. P. Lei C. L. (2011). Pupation behaviour, depth, and site of *Spodoptera exigua*. Bull. Insectol. 64, 209–214.

