# Supplementary material for: Caterpillar-Induced Volatile Emissions in Cotton: The Relative Importance of Damage and Insect-Derived Factors
Source: Front Plant Sci. 2021 Aug 3;12:709858. doi: 10.3389/fpls.2021.709858 (PMC8369242; doi:10.3389/fpls.2021.709858)
Supplement: Supplementary file 1 [file Data_Sheet_1.docx]

Supplementary Material

1. **Supplementary Figures and Tables**

**1.1 Supplementary Tables**

**Supplementary Table 1.** Local emission rate of volatiles (mean ±SE) emitted (ng/hour) by cotton plants (*n* = 4-6) induced by mechanical damage (MD), mechanical damage and regurgitant from *Spodoptera exigua* fed on cotton leaves (MD+CR) and mechanical damage and regurgitant from *S. exigua* fed on maize leaves (MD+MR). Volatiles were collected after 2-hours of induction. Statistically differences among treatments are indicated by different letters for each compound (Anova followed by FDR-corrected post-hoc tests, *P* < 0.05).

|  | **Local induction - 2h** | | | |  |
| --- | --- | --- | --- | --- | --- |
| **Compound** | **Control** | **MD** | **MD+CR** | **MD+MR** | ***P* value** |
| 4-hexen-1-ol, acetate | 0 ± 0 **a** | 6.44 ± 1.61 **ab** | 12.10 ± 2.88 **b** | 12.51 ± 2.79 **b** | **0.001** |
| α -pinene | 1.93 ± 1.16 **a** | 17.03 ± 3.81 **b** | 16.59 ± 5.29 **b** | 18.11 ± 3.96 **b** | **0.01** |
| Beta pinene | 0.19 ± 0.21 a | 1.58 ± 0.97 a | 4.81 ± 1.77 a | 3.30 ± 1.55 a | 0.07 |
| β -myrcene | 0.82 ± 0.60 a | 6.46 ± 1.98 a | 14.10 ± 6.92 a | 9.37 ± 2.51 a | 0.09 |
| β -ocimene | 0 ± 0 a | 1.55 ± 0.97 a | 2.66 ± 2.66 a | 0 ± 0 a | 0.44 |
| DMNT | 0 ± 0 | 0 ± 0 | 0 ± 0 | 0 ± 0 | - |
| TMTT | 0 ± 0 | 0 ± 0 | 0 ± 0 | 0 ± 0 | - |
| Caryophyllene | 4.38 ± 2.15 a | 9.00 ± 3.44 a | 13.05 ± 4.16 a | 10.33 ± 2.58 a | 0.25 |
| Humulene | 0.91 ± 0.42 a | 2.83 ± 0.87 a | 3.18 ± 1.50 a | 2.01 ± 0.90 a | 0.34 |
| Indole | 0 ± 0 | 0 ± 0 | 0 ± 0 | 0 ± 0 | - |

**Supplementary Table 2.** Local emission rate of volatiles (mean ±SE) emitted (ng/hour) by cotton plants (*n* = 4-6) induced by mechanical damage (MD), mechanical damage and regurgitant from *Spodoptera exigua* fed on cotton leaves (MD+CR) and mechanical damage and regurgitant from *S. exigua* fed on maize leaves (MD+MR). Volatiles were collected after 24-hours of induction. Statistically differences among treatments are indicated by different letters for each compound (Anova followed by FDR-corrected post-hoc tests, *P* < 0.05).

|  | **Local induction - 24h** | | | |  |
| --- | --- | --- | --- | --- | --- |
| **Compound** | **Control** | **MD** | **MD+CR** | **MD+MR** | ***P* value** |
| 4-hexen-1-ol, acetate | 0 ± 0 a | 3.95 ± 1.09 a | 12.24 ± 6.24 a | 12.87 ± 5.16 a | 0.07 |
| α -pinene | 1.93 ± 1.16 **a** | 9.81 ± 3.02 **b** | 18.40 ± 5.32 **b** | 20.85 ± 1.04 **bc** | **0.001** |
| Beta pinene | 0.19 ± 0.21 **a** | 0.56 ± 0.56 **a** | 4.65 ± 0.53 **b** | 4.40 ± 0.31 **b** | **0.001** |
| β -myrcene | 0.82 ± 0.60 **a** | 5.49 ± 1.90 **a** | 13.36 ± 3.43 **b** | 15.28 ± 2.51 **b** | **0.001** |
| β -ocimene | 0 ± 0 a | 2.12 ± 2.12 a | 2.13 ± 2.13 a | 3.0 ± 1.26 a | 0.54 |
| DMNT | 0 ± 0 | 0 ± 0 | 0 ± 0 | 0 ± 0 | - |
| TMTT | 0 ± 0 | 0 ± 0 | 0 ± 0 | 0 ± 0 | - |
| Caryophyllene | 4.38 ± 2.15 a | 7.50 ± 3.47 a | 8.52 ± 2.09 a | 10.27 ± 2.14 a | 0.38 |
| Humulene | 0.91 ± 0.42 a | 1.12 ± 0.47 a | 2.13 ± 0.73 a | 2.47 ± 0.79 a | 0.21 |
| Indole | 0 ± 0 | 0 ± 0 | 0 ± 0 | 0 ± 0 | - |

**Supplementary Table 3.** Local emission rate of volatiles (mean ±SE) emitted (ng/hour) by cotton plants (*n* = 4-6) induced by mechanical damage (MD), mechanical damage and regurgitant from *Spodoptera exigua* fed on cotton leaves (MD+CR) and mechanical damage and regurgitant from *S. exigua* fed on maize leaves (MD+MR). Volatiles were collected after 48-hours of induction. Statistically differences among treatments are indicated by different letters for each compound (Anova followed by FDR-corrected post-hoc tests, *P* < 0.05).

|  | **Local induction - 48h** | | | |  |
| --- | --- | --- | --- | --- | --- |
| **Compound** | **Control** | **MD** | **MD+CR** | **MD+MR** | ***P* value** |
| 4-hexen-1-ol, acetate | 0 ± 0 **a** | 3.54 ± 0.96 **a** | 14.50 ± 6.10 **b** | 4.80 ± 1.15 **a** | **0.01** |
| α -pinene | 1.93 ± 1.16 **a** | 9.95 ± 1.19 **b** | 20.87 ± 3.51 **c** | 10.76 ± 2.11 **b** | **0.001** |
| Beta pinene | 0.19 ± 0.21 a | 0.90 ± 0.57 a | 3.17 ± 1.04 a | 2.40 ± 1.21 a | 0.07 |
| β -myrcene | 0.82 ± 0.60 **a** | 3.83 ± 1.16 **a** | 10.81 ± 1.27 **b** | 6.67 ± 1.16 **a** | **0.001** |
| β -ocimene | 0 ± 0 **a** | 0 ± 0 **a** | 7.17 ± 4.14 **ab** | 0.88 ± 0.59 **a** | **0.05** |
| DMNT | 0 ± 0 **a** | 2.28 ± 1.40 **a** | 10.34 ± 1.67 **bc** | 5.47 ± 2.26 **ab** | **0.001** |
| TMTT | 0 ± 0 a | 2.32 ± 1.55 a | 11.82 ± 6.43 a | 13.51 ± 7.22 a | 0.13 |
| Caryophyllene | 4.38 ± 2.15 a | 6.79 ± 1.41 a | 9.85 ± 1.37 a | 6.11 ± 2.15 a | 0.27 |
| Humulene | 0.91 ± 0.42 a | 1.77 ± 0.55 a | 2.28 ± 0.75 a | 1.40 ± 0.67 a | 0.44 |
| Indole | 0 ± 0 **a** | 0.71 ± 0.32 **a** | 8.52 ± 2.21 **b** | 2.58 ± 0.43 **a** | **0.001** |

**Supplementary Table 4.** Systemic emission rate of volatiles (mean ±SE) emitted (ng/hour) by cotton plants (*n* = 4-6) induced by mechanical damage (MD), mechanical damage and regurgitant from *Spodoptera exigua* fed on cotton leaves (MD+CR) and mechanical damage and regurgitant from *S. exigua* fed on maize leaves (MD+MR). Volatiles were collected after 48-hours of induction. Statistically differences among treatments are indicated by different letters for each compound (Anova followed by FDR-corrected post-hoc tests, *P* < 0.05).

| **Systemic induction - 48 h** | | | | | |
| --- | --- | --- | --- | --- | --- |
| **Compound** | **Control** | **MD** | **MD + CR** | **MD + MR** | ***P* value** |
| α -pinene | 5.17 ± 1.78 **a** | 7.86± 0.91 **a** | 25.72 ± 2.45 **b** | 9.84 ± 1.71 **a** | **0.001** |
| β -pinene | 0 ± 0 **a** | 5.59 ± 0.82 **a** | 5.10 ± 0.96 **a** | 10.53 ± 3.47 **b** | **0.006** |
| β -myrcene | 0 ± 0 **a** | 0.23 ± 0.07 **a** | 8.33 ± 3.01 **b** | 1.36 ± 0.18 **a** | **0.002** |
| D -limonene | 0 ± 0 **a** | 6.66 ± 3.53 **b** | 17.94 ± 2.24 **c** | 13.16 ± 2.05 **c** | **0.001** |
| β -ocimene | 0 ± 0 **a** | 0.78 ± 0.30 **a** | 12.51 ± 2.99 **b** | 9.00 ± 4.46 **b** | **0.007** |
| Linalool | 0 ± 0 | 1.42 ± 0.58 | 4.46 ± 1.69 | 6.60 ± 3.11 | 0.07 |
| Caryophyllene | 2.86 ± 1.37 | 5.67 ± 2.38 | 4.05 ± 0.43 | 6.90 ± 3.18 | 0.55 |
| (*E*)-β -farnesene | 0 ± 0 **a** | 0.07 ± 0.02 **a** | 51.95 ± 21.22 **b** | 2.32 ± 2.03 **a** | **0.005** |

**Supplementary Table 5.** Emission rate of volatiles (mean ±SE) emitted (ng/hour) by cotton plants incubated in water (Control), cotton plants incubated in a regurgitant from *Spodoptera exigua* fed on cotton leaves (Cotton R) and cotton plants incubated in a regurgitant from *S. exigua* fed on maize leaves (Maize R) (*n* = 4-6). Volatiles were collected after 2-, 24- and 48-hours of induction. Statistically differences among treatments are indicated by different letters within each time point for each compound (Anova followed by FDR-corrected post-hoc tests, P < 0.05).


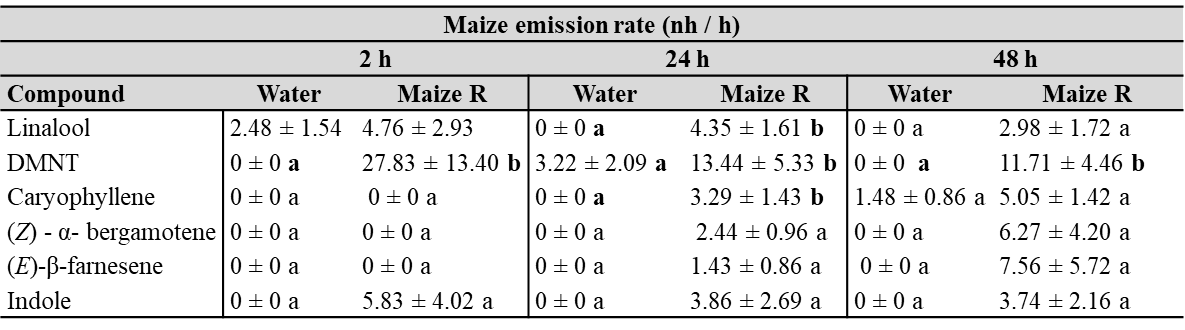


**Supplementary Table 6.** Emission rate of volatiles (mean ±SE) emitted (ng/hour) by maize plants incubated in water (Control) and maize plants incubated in a regurgitant from *Spodoptera exigua* fed on maize leaves (Maize R) (*n* = 4-6). Volatiles were collected after 2-, 24- and 48-hours of induction. Statistically differences among treatments are indicated by different letters within each time point for each compound (Anova followed by FDR-corrected post-hoc tests, *P* < 0.05).

**1.2 Supplementary Figures**

**A B**

**
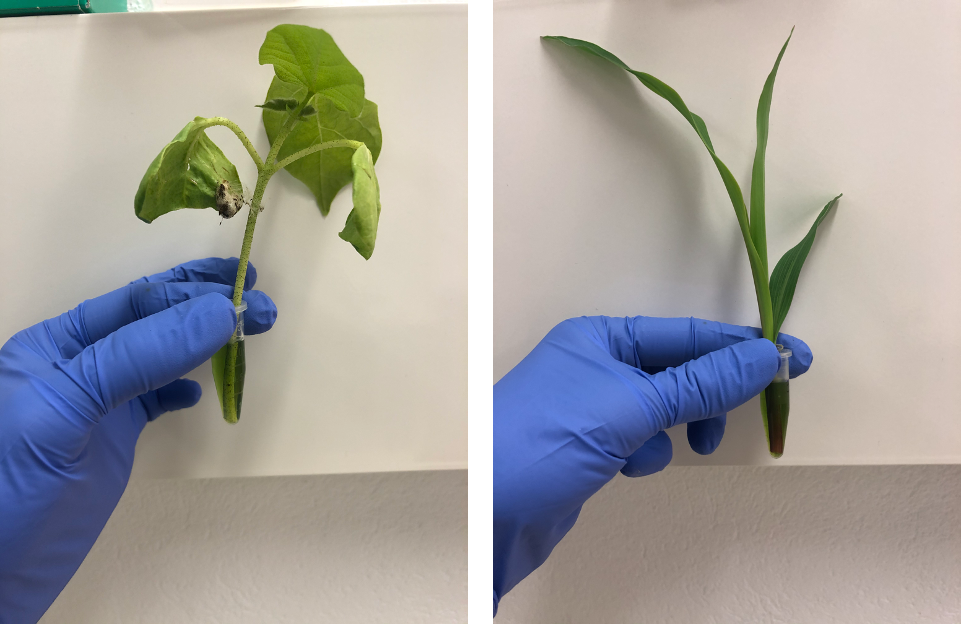
**

**Supplementary Figure 1.** Incubation experimental setup. (**A**) Cotton plants incubated in regurgitant solution after 48 hours. (**B**) Maize plants incubated in regurgitant solution after 48 hours.

**
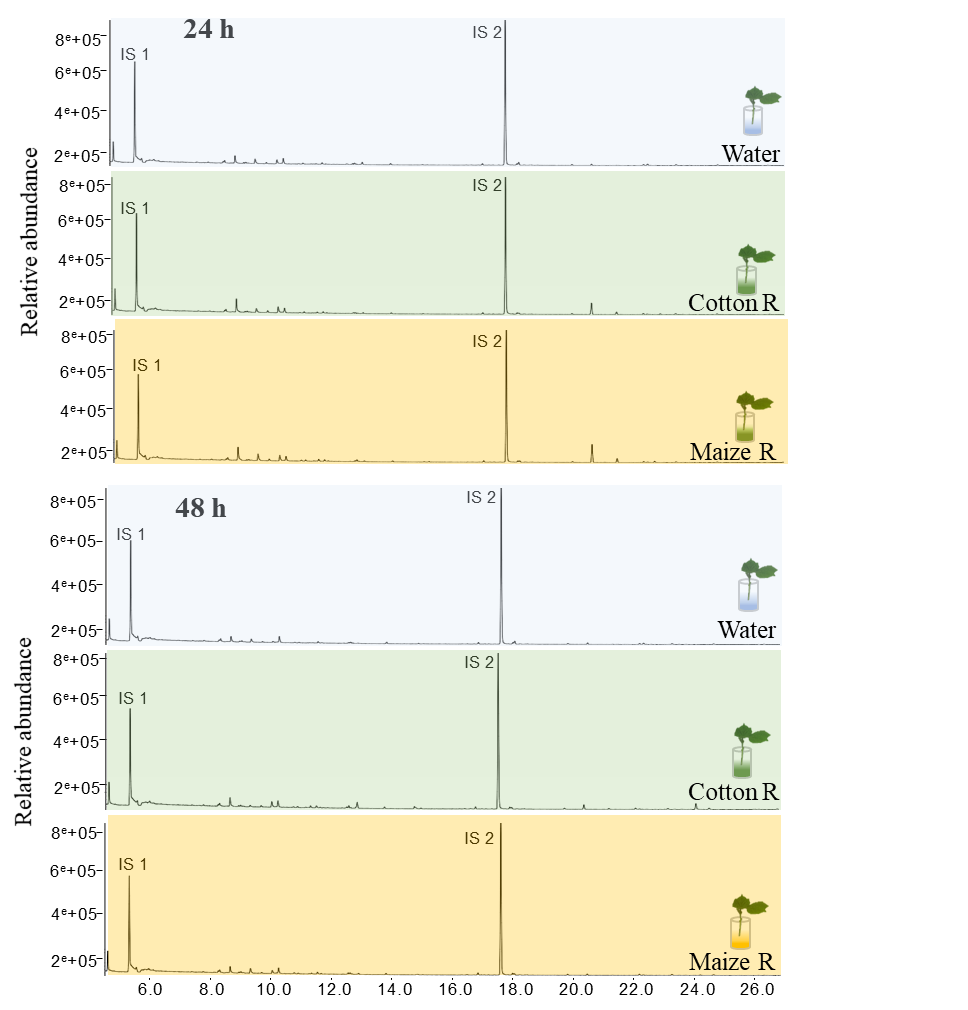
**

**Supplementary Figure 2.** Typical chromatograms of volatiles emitted by cotton plants incubated in water, regurgitant solution of cotton-fed *S. exigua* (Cotton R) and regurgitant solution of maize-fed *S. exigua* (Maize R) after 24 and 48 hours of incubation. IS1 and IS2 are the internal standards: *n*-octane and nonyl acetate, respectively.


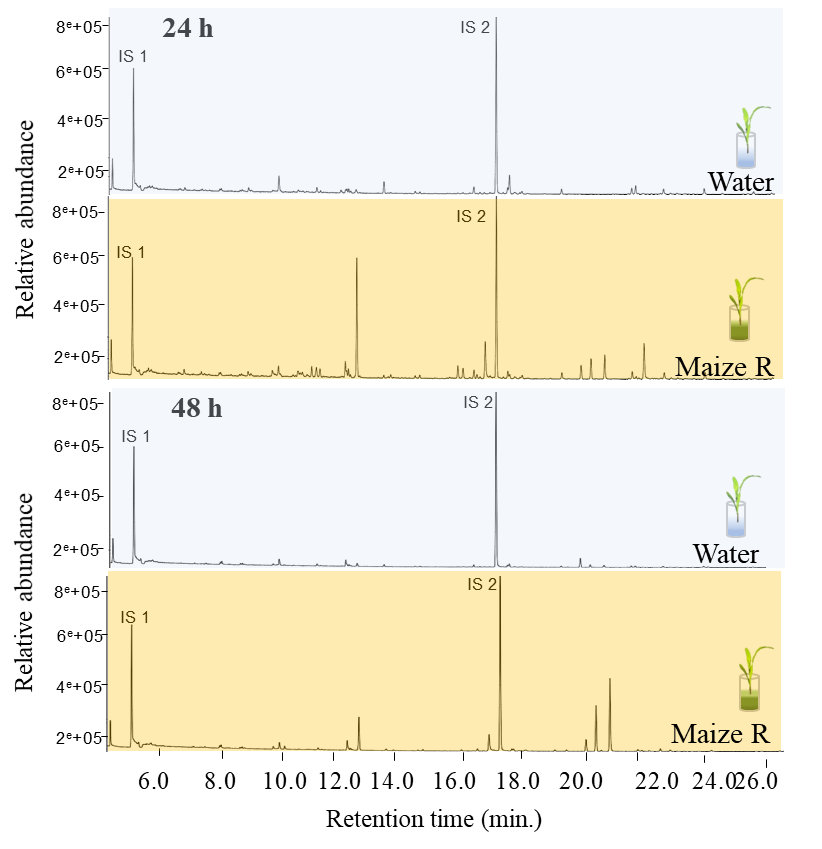


**Supplementary Figure 3.** Typical chromatograms of volatiles emitted by maize plants incubated in water and regurgitant solution of maize-fed *S. exigua* (Maize R) after 24 and 48 hours of incubation. IS1 and IS2 are the internal standards: *n*-octane and nonyl acetate, respectively.
